# Transmission and Drug Resistance Genotype of Multidrug-Resistant or Rifampicin-Resistant Mycobacterium tuberculosis in Chongqing, China

**DOI:** 10.1128/spectrum.02405-21

**Published:** 2022-10-10

**Authors:** Bing Zhao, Chunfa Liu, Jiale Fan, Aijing Ma, Wencong He, Yan Hu, Yanlin Zhao

**Affiliations:** a National Center for Tuberculosis Control and Prevention, Chinese Center for Disease Control and Prevention, Changping, Beijing, China; b National Institute for Communicable Disease Control and Prevention, Chinese Center for Disease Control and Prevention, Changping, Beijing, China; c Tuberculosis Reference Laboratory, Chongqing Tuberculosis Control Institute, Jiulongpo, Chongqing, China; Francis Crick Institute

**Keywords:** MDR/RR-TB, recent transmission, drug resistance, WGS

## Abstract

Multidrug-resistant or rifampicin-resistant tuberculosis (MDR/RR-TB) is a global barrier for the Stop TB plan. To identify risk factors for treatment outcome and cluster transmission of MDR/RR-TB, whole-genome sequencing (WGS) data of isolates from patients of the Chongqing Tuberculosis Control Institute were used for phylogenetic classifications, resistance predictions, and cluster analysis. A total of 223 MDR/RR-TB cases were recorded between 1 January 2018 and 31 December 2020. Elderly patients and those with lung cavitation are at increased risk of death due to MDR/RR-TB. A total of 187 MDR/RR strains were obtained from WGS data; 152 were classified as lineage 2 strains. Eighty (42.8%) strains differing by a distance of 12 or fewer single nucleotide polymorphisms were classified as 20 genomic clusters, indicating recent transmission. Patients infected with lineage 2 strains or those with occupations listed as “other” are significantly associated with a transmission cluster of MDR/RR-TB. Analysis of resistant mutations against first-line tuberculosis drugs found that 76 (95.0%) of all 80 strains had the same mutations within each cluster. A total of 55.0% (44 of 80) of the MDR/RR-TB strains accumulated additional drug resistance mutations along the transmission chain, especially against fluoroquinolones (63.6% [28 of 44]). Recent transmission of MDR/RR strains is driving the MDR/RR-TB epidemics, leading to the accumulation of more serious resistance along the transmission chains.

**IMPORTANCE** The drug resistance molecular characteristics of MDR/RR-TB were elucidated by genome-wide analysis, and risk factors for death by MDR/RR-TB were identified in combination with patient information. Cluster characteristics of MDR/RR-TB in the region were analyzed by genome-wide analysis, and risk factors for cluster transmission (recent transmission) were analyzed. These analyses provide reference for the prevention and treatment of MDR/RR-TB in Chongqing.

## INTRODUCTION

Multidrug-resistant or rifampicin (RIF)-resistant tuberculosis (MDR/RR-TB; resistance to at least rifampicin and/or isoniazid [INH]) represents a major threat to public health worldwide (1). The treatment period of patients with MDR/RR-TB is longer than that of patients with drug-susceptible TB, based on less effective and more toxic drugs, and the cure rate decreases to 60% on a global level (2). In patients with drug-susceptible pulmonary TB, the standard 6-month treatment regimen (2HRZE/4HR) is used. In MDR/RR-TB patients on longer regimens, fluoroquinolones (FQs) (including levofloxacin [LVX] and moxifloxacin [MXF]), bedaquiline (BDQ), linezolid (LZD), and at least one of clofazimine (CFZ) and cycloserine or terizidone should be used to ensure that treatment starts with at least four TB agents and that at least three agents are included for the rest of the treatment after BDQ is stopped. If only one or two of FQs, BDQ, and LZD are used, both CFZ and cycloserine or terizidone need to be included. If the treatment regimen cannot be composed of the agents listed above, all other medicines that can be used are added to complete it (3, 4). To identify the risk factors for treatment outcome of MDR/RR-TB is beneficial to treatment of this population. We employed the sociodemographic and clinical characteristics of patients in Chongqing, China, to analyze the risk factors.

China has the second highest incidence of MDR/RR-TB, with an estimate of 65,000 MDR/RR-TB patients in 2019, which accounts for 14% of all of the MDR/RR-TB burden in the world (4). MDR/RR-TB may result from recent infection with an MDR/RR strain (recent transmission) or from initial transmissions of a non-MDR/RR strain that developed to MDR/RR-TB after many years (5). A previous study showed that the transmission of MDR-TB was attributed to most MDR prevalence in high-burden settings. Merely improving the drug-susceptible TB treatment is unlikely to effectively reduce the future incidence of MDR-TB (6). Therefore, tracking recent transmission is an essential action to control widespread TB. To analyze the recent transmission of MDR/RR-TB in Chongqing, we isolated the strains from patients in the past 2 years.

By clustering strains based on genotype similarities, multiple analysis methods are performed to detect recent transmission chains, including IS*6110* DNA fingerprinting (7), spoligotyping (8), variable-number tandem repeats of mycobacterial interspersed repetitive units (MIRU-VNTR) (9), and whole-genome sequencing (WGS)-based genotyping (10) or core genome multilocus sequence typing (cg-MLST) (11), which is the most common method. Research has shown that spoligotyping could encompass transmission events that occurred almost 200 years prior to sampling, while 24-locus MIRU-VNTR often represented 3 decades of transmission. Instead, WGS-based genotyping applying low single nucleotide polymorphisms (SNPs) or cg-MLST allele thresholds allows for determination of recent transmission events in recent years (12). A retrospective observational study using WGS and epidemiological investigation has proved that recent transmission of MDR-TB strains drives the MDR-TB epidemic in Shanghai, China (13). This research suggested that it is a priority to halt ongoing transmission of MDR strains for TB control in China. A pilot study in Europe using a WGS-based approach (cg-MLST) showed that the WGS-based surveillance can efficiently elucidate the dynamics of in-country and cross-border MDR/RR-TB transmission (14). Therefore, we sequenced all the MDR/RR strains in Chongqing and used the cg-MLST approach to analyze the SNP distance.

Besides the identification of transmission clusters, WGS has been used clinically to diagnose drug resistance based on known resistance of Mycobacterium tuberculosis, especially for first-line tuberculosis drugs (15–17). Several web tools for detection of genotype SNPs and indels in Mycobacterium tuberculosis have been developed to predict resistance. It is convenient and possible to predict drug resistance based on the drug resistance-related mutations database, such as Mykrobe and TB-Profiler (18). We compared these tools for prediction of drug resistance using MDR/RR strains. The resistance mutations along the cluster transmission were analyzed to detect if there is accumulation of resistance mutations.

Chongqing is a municipality in the southwest of China with about 32 million people. It has a reasonably well-functioning tuberculosis control program, including participation in the Bill and Melinda Gates Foundation and the Global Fund in 2009 and 2013 (19). An integrated model has been implemented since 2016; in this model, the Chinese Center for Disease Control and Prevention (CDC) handles the planning, supervision, and health education of TB control, appointed hospitals are responsible for TB diagnosis and treatment, and primary health care deals with case management and patient referrals (20). Recognizing the burden of recent transmission for the TB epidemic will further improve the disease control program. Our observational retrospective study tried to improve surveillance of recent transmission and drug genotypic resistance patterns of MDR/RR-TB using WGS in Chongqing. Our research may promote MDR/RR-TB control in Chongqing and other similar regions.

## RESULTS

### Treatment results of MDR/RR-TB patients.

From 1 January 2018 to 31 December 2020, we diagnosed 223 (24.6%) MDR/RR-TB patients by phenotypic drug susceptibility testing (DST) from a total 906 culture-positive TB patients in the Chongqing Tuberculosis Control Institute. Treatment information for 200 MDR/RR-TB patients with culture-positive isolates was collected. As shown in Table 1, except for 2 patients who died from other causes, 23 (11.5%) patients died from MDR/RR-TB, and 80 (40%) patients received favorable outcomes (34 cured and 46 completed the treatment). Ninety-five (47.5%) patients were classified into the pending group for various reasons, with 49 (24.5%) still undergoing treatment, while the reasons for the other 46 cases include loss to follow-up, severe adverse drug reactions, and treatment refusal.

**TABLE 1:** Treatment results in MDR/RR-TB patients

Treatment result	No. (%) of cases (*n* = 200)	Subgroup	No. (%) of cases (*n* = 200)
Favored outcome	80 (40.0)	Cured	34 (17.0)
		Completed	46 (23.0)
Pending	95 (47.5)	Treatment refusal	24 (12.0)
		In treatment	49 (24.5)
		Lost to follow-up	14 (7.0)
		Severe adverse drug reactions	6 (3.0)
		Others	2 (1.0)
Died	23 (11.5)	Died	23 (11.5)
Died from other causes	2 (1.0)	Died from other causes	2 (1.0)

Sociodemographic characteristics (gender, age, residence, occupation, etc.) and clinical characteristics (complications, previous TB treatment history etc.) of patients in those groups from Table 1 (excluding the 2 deaths from other causes) are shown in Table 2. Univariate and logistic regression analyses were also performed for these 198 patients. As shown in Table 3, the results indicated that age, occupation, and lung cavitation were significant between favorable outcome and death (*P* ≤ 0.1).

**TABLE 2:** Characteristics of patients with MDR/RR-TB

Characteristic	Total (*n* = 200)		Favored outcome (*n* = 80)		Pending (*n* = 95)		Died (*n* = 23)	
	No.	%	No.	%	No.	%	No.	%
Gender								
Male	148	74.0	59	40.1	71	48.3	17	11.6
Female	52	26.0	21	41.2	24	47.1	6	11.8
Age, yr								
15–34	48	24.0	28	58.3	17	35.4	1	2.1
35–54	97	48.5	30	30.9	59	60.8	8	8.2
≥55	55	27.5	22	40.0	19	34.5	14	26.4
Occupation								
Others	59	29.5	24	40.7	31	52.5	3	5.1
Farmer	115	57.5	44	38.6	50	43.9	20	17.5
Worker	26	13.0	12	46.2	14	53.8	0	0
Residence								
Rural	116	58.0	51	44.3	46	40.0	18	15.7
Urban	84	42.0	29	34.9	49	59.0	5	6.0
Category								
Migrant	25	12.5	9	36.0	15	60.0	1	4.0
Resident	175	87.5	71	41.0	80	46.2	22	12.7
Type of patient								
Previously treated	152	76.0	60	40.0	71	47.3	19	12.7
New	48	24.0	20	41.7	24	50.0	4	8.3
Lung cavitation								
No	119	59.5	54	45.4	54	45.4	11	9.2
Yes	81	40.5	26	32.9	41	51.9	12	15.2
Diabetes mellitus								
No	189	94.5	74	39.4	93	49.5	21	11.2
Yes	11	5.5	6	60.0	2	20.0	2	20.0
Diagnosis delay (days)								
Above mean	42	21.0	15	35.7	21	50.0	6	14.3
Below mean	158	79.0	65	41.1	74	46.8	17	10.8
Drug susceptibility								
MDR/RR-TB	103	51.5	41	39.8	53	51.5	9	8.7
Pre-XDR-TB	92	46.0	37	41.1	39	43.3	14	15.6
XDR-TB	5	2.5	2	40.0	3	60.0	0	0
History of treatments								
No drugs	15	7.5	10	66.7	4	26.7	1	6.7
First-line drugs only	127	63.5	53	42.1	57	45.2	16	12.7
Second-line drugs	58	29.0	17	29.8	34	59.6	6	10.5

**TABLE 3:** Univariate analysis of the risk factor for treatment outcome^*a*^

Characteristic	A vs B, *χ*^2^	*P* value	A vs C, *χ*^2^	*P* value	B vs C, *χ*^2^	*P* value
Sex	0.022	0.882	<0.001	0.987	0.007	0.935
Age (yr)	11.154	0.004	11.688	0.003	15.722	<0.001
Occupation	0.143	0.931	8.345	0.015	9.533	0.009
Residence	4.131	0.042	1.701	0.192	6.643	0.010
Category	0.756	0.384	0.971	0.324	2.068	0.150
Type of patient	0.002	0.968	0.579	0.447	0.643	0.426
Lung cavitation	2.088	0.148	2.970	0.085	0.608	0.435
Diabetes mellitus	1.793	0.181^*b*^	0.036	0.850	2.456	0.117
Drug susceptibility	0.503	0.778	1.887	0.389	3.323	0.190
History of treatments	7.150	0.028	1.328	0.515	0.789	0.674
Diagnosis delay (days)	0.299	0.584	0.592	0.441	0.166	0.683

a A, favored outcome group; B, pending group; C, died.

b *P* value was calculated by continuity correction.

Next, these variables were progressed to the multivariate analysis (Table 4). Age was the risk factor for death for elderly patients. Between the pending and death groups, the results from the univariate analysis indicated that age, occupation, and residence were significant risk factors (*P* ≤ 0.1). These variables were progressed to the multivariate analysis (Table 4). The age was the risk factor for older patients. Between the favorable outcome and pending groups, the results from the univariate analysis indicated that age, residence, and history of treatments were significant (*P* ≤ 0.1). Then, these variables were progressed to the multivariate analysis (Table 4). History of treatments was the risk factor; the patients previously treated with second-line drugs were more likely to have pending outcome.

**TABLE 4:** Multivariate logistic regression of the risk factor for treatment outcome

Characteristic	OR^*a*^ (95% CI)	*P* value^*b*^
Age (yr)		
15–34	Ref.	
35–54	3.466 (1.590–7.558)	0.002
≥55	1.600 (0.647–3.960)	0.309
Residence		
Rural	Ref.	
Urban	1.819 (0.939–3.524)	0.076
History of treatments		
No drugs	Ref.	
First-line drugs only	2.065 (0.577–7.396)	0.265
Second-line drugs	3.906 (1.005–15.184)	0.049
Age (yr)		
15–34	Ref.	
35–54	4.995 (0.556–44.860)	0.151
≥55	12.923 (1.424–117.256)	0.023
Occupation		
Others	Ref.	
Farmer	2.493 (0.526–11.830)	0.250
Worker		0.999
Lung cavitation		
No	Ref.	
Yes	3.043 (0.978–9.473)	0.055
Age (yr)		
35–54	Ref.	
15–34	0.692 (0.074–6.464)	0.747
≥55	5.095 (1.770–14.670)	0.003
Occupation		
Others	Ref.	
Farmer	1.904 (0.287–12.634)	0.505
Worker		0.999
Residence		
Urban	Ref.	
Rural	1.485 (0.303–7.281)	0.626

a OR, odds ratio; Ref., reference.

b The first set of *P* values is for favored outcome versus pending, the second set is for favored outcome versus died, and the third is for pending versus died.

### Recent transmissions of MDR/RR-TB in Chongqing, China.

**FIG 1:**
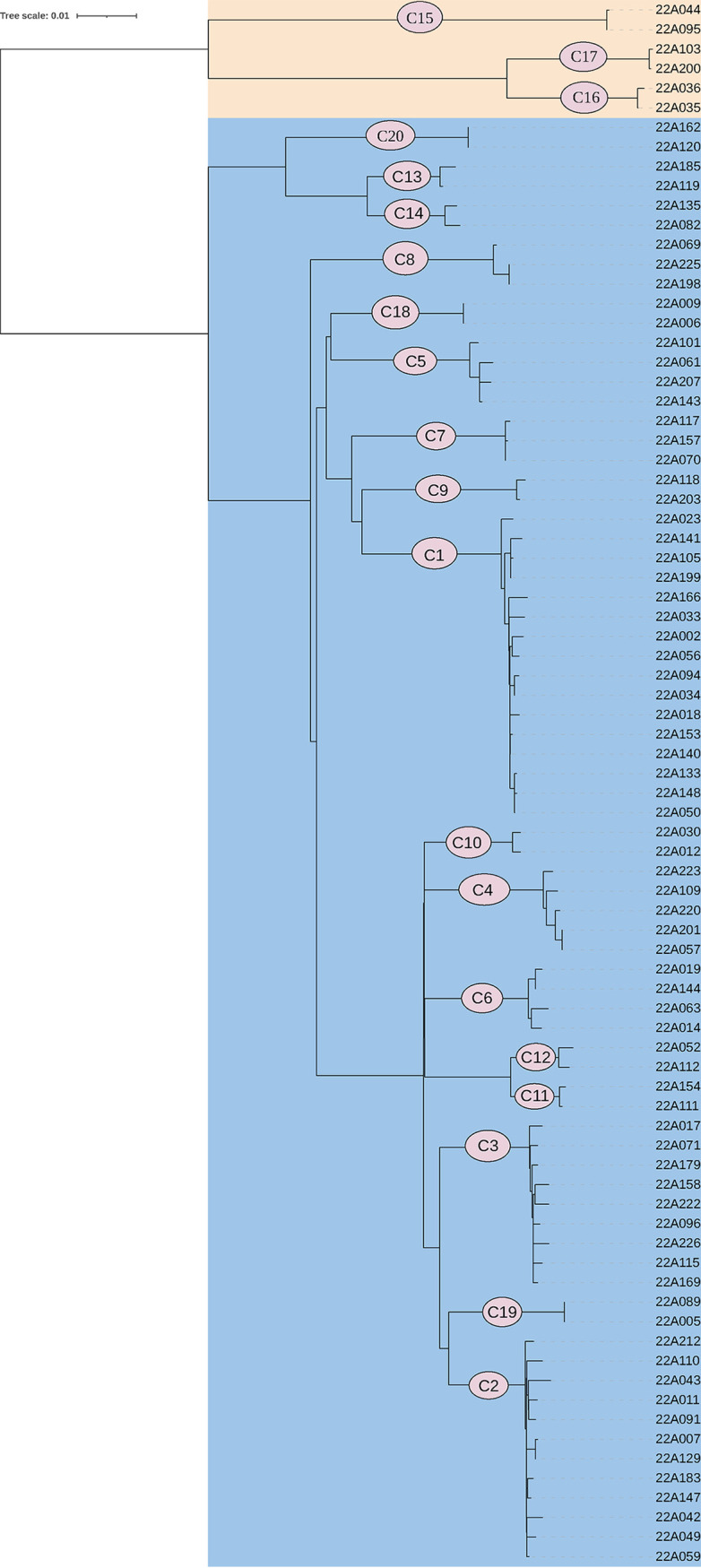
Minimum distance spanning tree (MST) of MDR/RR strains based on cg-MLST typing. Cluster strains that differed by 12 or fewer allele thresholds are shown by different indicators (C1 to C20, cluster 1 to cluster 20). The colors indicate different lineages (pink, lineage 4; blue, lineage 2).

**FIG 2:**
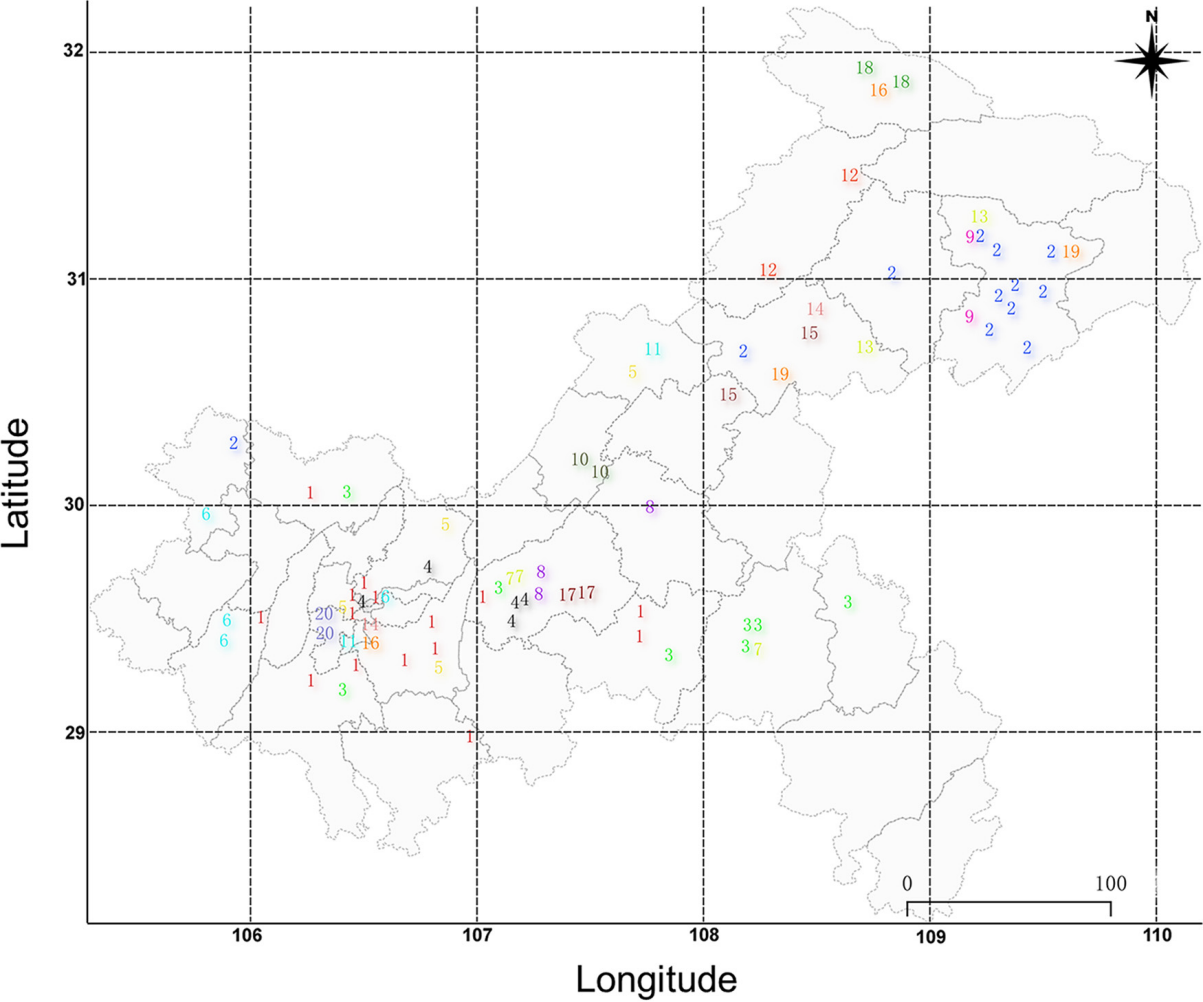
Spatial distribution of genetic cluster strains.

Incident TB is a combination of recent transmission and remote transmission (initial transmissions of a non-MDR/RR strain that developed into MDR/RR-TB over a period of years) (21). To evaluate the burden of recent transmission of MDR/RR-TB in Chongqing, a minimum distance spanning tree (MST) based on SNP distance was calculated on the basis of the WGS sequencing data. A total of 187 isolates were obtained from 200 culture-positive MDR/RR isolates. Eighty (42.8%) isolates differed in 20 genomic clusters by an allele cutoff of 12 or fewer based on the cg-MLST approach (see Fig. S1 in the supplemental material). The clusters, ranging from 2 patients (12 clusters) to 16 patients (1 cluster), presented with recent transmission (Fig. 1). Spatial-location analysis revealed that 43 (53.8%) clustered isolates were located in the same district (Fig. 2). Pearson correlation analysis demonstrated minimal association between spatial distance and genetic distance (SNPs) for all 80 genomic clustered strains (CRR = −0.09; *P* = 0.14). Only in cluster 4, a strong positive correlation (CRR) was observed between spatial distance and genetic distance (SNPs) (CRR = 0.95; *P* < 0.01). (Details are shown in File S1.)

Of all the MDR/RR isolates, 152 (85.4%) were grouped into lineage 2. As shown in Fig. 1, within the 80 isolates in 20 genomic clusters, 74 isolates (17 clusters) were lineage 2 and 6 isolates (3 clusters) were lineage 4. Then, we analyzed the risk factors for recent transmission. The results of univariate analysis suggested that gender, occupation, residence, and lineage were significantly associated with disease transmission (*P* ≤ 0.1) (Table 5). Then, gender, occupation, residence, and lineage were progressed to multivariate analysis (Table 6). Occupation and lineage were the risk factors by which the patients with other occupations (mainly household management service industry) or infected by lineage 2 strains were more likely to be part of a cluster. The risk factors of patients who were infected by lineage 2 were analyzed (data not shown). Cluster transmission was the only risk factor for Mycobacterium tuberculosis lineage 2 infection. In conclusion, cluster transmission of lineage 2 strains by patients with other occupations is a significant risk factor driving the epidemic toward MDR/RR-TB.

**TABLE 5:** Univariate analysis of the risk factor for recent transmission among patients

Factor	Noncluster (*n* = 107)		Cluster (*n* = 80)		*χ* ^2^	*P* value
	No.	%	No.	%		
Gender					2.780	0.095
Male	85	60.7	55	39.3		
Female	22	46.8	25	53.2		
Age, yr					4.125	0.125
≥55	35	66.0	18	34.0		
35–54	52	57.1	39	42.9		
15–34	20	46.5	23	53.5		
Occupation					12.159	0.002
Farmer	72	66.7	36	33.3		
Worker	15	57.7	11	42.3		
Others	20	37.7	33	62.3		
Residence					7.584	0.006
Rural	71	65.7	37	34.3		
Urban	36	45.6	43	54.4		
Category					0.036	0.850
Migrant	13	59.1	9	40.9		
Resident	94	57.0	71	43.0		
Type of patient					2.118	0.146
Previously treated	86	60.1	57	39.9		
New	21	47.7	23	52.3		
Lung cavitation					0.107	0.743
Yes	44	58.7	31	41.3		
No	63	56.3	49	43.8		
Diabetes mellitus					0.225	0.635
No	102	57.6	75	42.4		
Yes	5	50.0	5	50.0		
Diagnosis delay					0.062	0.803
Above mean	23	59.0	16	41.0		
Below mean	84	56.8	64	43.2		
Lineage					11.562	0.001
Lineage 2	78	51.3	74	48.7		
Lineage 4	29	82.9	6	17.1		
History of treatments					0.106	0.948
No drugs	8	53.3	7	46.7		
First-line drugs only	67	57.8	49	42.2		
Second-line drugs	32	57.1	24	42.9		
Treatment results					4.325	0.228
Favored outcome	39	51.3	37	48.7		
Pending	55	63.2	32	36.8		
Died	11	50.0	11	50.0		
Died from other causes	2	100.0	0	0.0		

**TABLE 6:** Multivariate logistic regression of the risk factor for recent transmission among patients

Characteristic	OR (95% CI)	*P* value
Gender		
Male	Ref.	
Female	1.590 (0.765–3.303)	0.214
Occupation		
Worker	Ref.	
Farmer	1.082 (0.323–3.623)	0.898
Others	2.890 (1.053–7.927)	0.039
Residence		
Rural	Ref.	
Urban	1.394 (0.524–3.706)	0.506
Lineage		
Lineage 4	Ref.	
Lineage 2	5.012 (1.896–13.247)	0.001

### Drug resistance profile of MDR/RR-TB.

**FIG 3:**
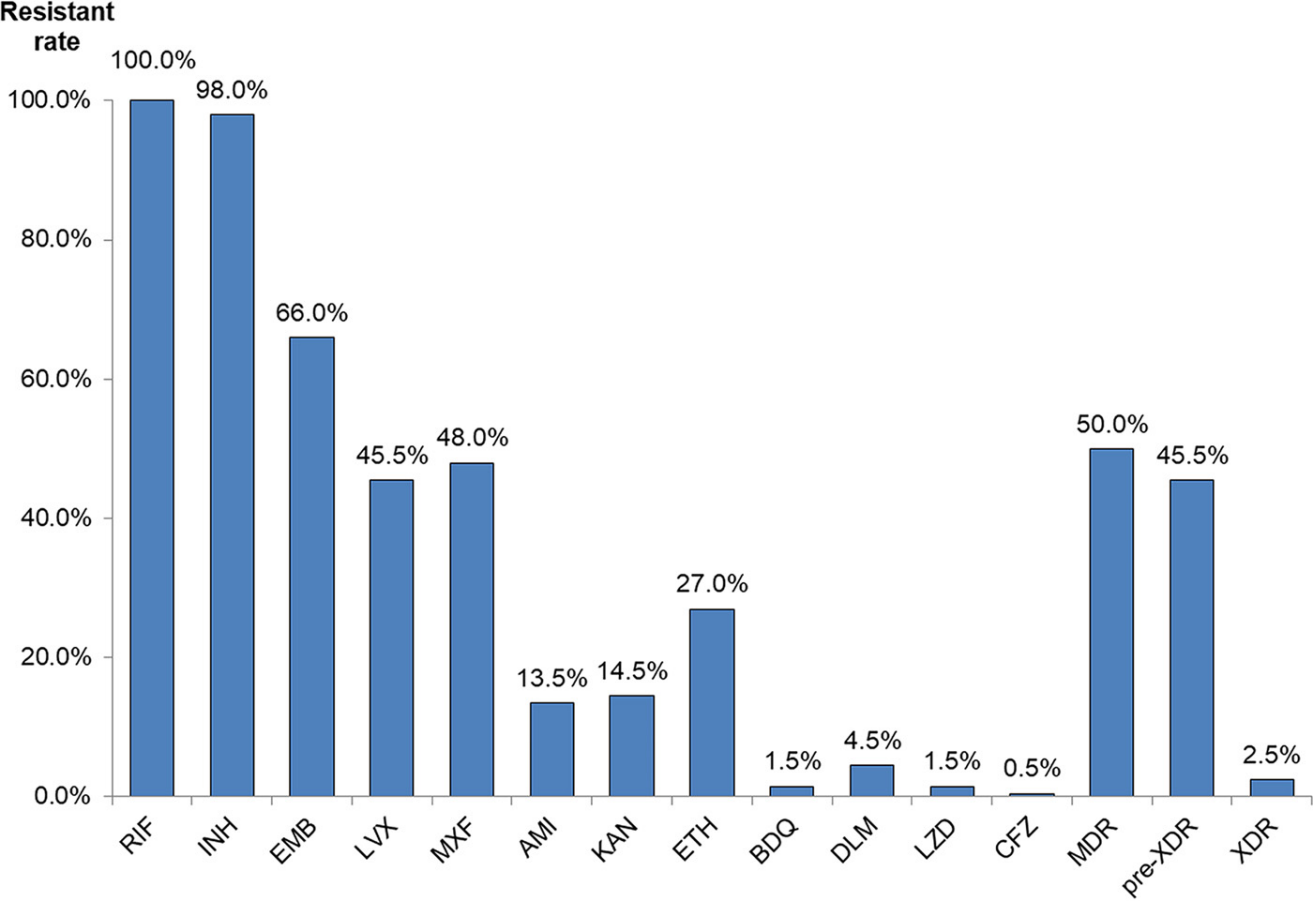
Phenotypic resistance rate of MDR/RR strains in Chongqing, China. RIF, rifampicin; INH, isoniazid; EMB, ethambutol; LVX, levofloxacin; MXF, moxifloxacin; AMI, amikacin; KAN, kanamycin; ETH, ethionamide; BDQ, bedaquiline; DLM, delamanid; LZD, linezolid; CFZ, clofazimine; MDR, multidrug resistant; pre-XDR, pre-extensively drug resistant; XDR, extensively drug resistant.

For all 200 culture-positive MDR/RR isolates, we calculated the rates of resistance to 12 antibiotics (Fig. 3; MIC details are in File S3). One hundred (50.0%) strains were MDR strains that were resistant to isoniazid (INH) and rifampicin (RIF). Ninety-one (45.5%) MDR/RR-TB cases were classified as pre-extensively drug resistant (pre-XDR), which are additionally resistant to one of the fluoroquinolones (FQs) (22). One hundred thirty-two (66.0%) strains were resistant to ethambutol (EMB), which is one of four first-line antituberculosis drugs. The rates of resistance to two FQs, LVX and MXF, were 45.5% and 48.0%. The rates of resistance to two aminoglycosides (AGs), kanamycin (KAN) and amikacin (AMK), were 14.5% and 13.5%. For new antituberculosis drugs, the rates of resistance to BDQ, delamanid (DLM), LZD, and CFZ were 1.5%, 4.5%, 1.5%, and 0.5%, respectively. Five (2.5%) MDR/RR-TB cases were classified as extensively drug resistant tuberculosis (XDR-TB), which, by definition, is resistant to a fluoroquinolone and either BDQ or LZD (or both) (22). Lineage 2 was more likely to be resistant to ethambutol than lineage 4 (Table 7).

**TABLE 7:** Drug resistance profiles of MDR/RR-TB strains

Drug	Lineage 2 (*n* = 152)		Lineage 4 (*n* = 35)		*χ* ^2^	*P*
	No.	%	No.	%		
Rifampicin	152	100.0	35	100.0		
Isoniazid	150	98.7	34	97.1		0.465^*a*^
Ethambutol	111	73.0	12	34.3	18.967	<0.001
Moxifloxacin	71	46.7	17	48.6	0.040	0.842
Levofloxacin	71	46.7	12	34.3	1.779	0.182
Kanamycin	19	12.5	3	8.6		0.771^*a*^
Amikacin	20	13.2	2	5.7		0.380^*a*^
Ethionamide	44	28.9	5	14.3	3.163	0.075
Bedaquiline	3	2.0	0	0.0		1.000^*a*^
Delamanid	7	4.6	1	2.9		1.000^*a*^
Linezolid	2	1.3	0	0.0		1.000^*a*^
Clofazimine	1	0.7	0	0.0		1.000^*a*^

a *P* value was calculated by Fisher’s exact test.

The agreement rate of phenotypic and genotypic drug susceptibility testing for 187 isolates is shown in Table 8. We found an average concordance of 93.1% across all 12 drugs, ranging from 78.6% (ETH) to 98.9% (LZD and CFZ). Genetic variants of multiple genes associated with drug resistance were identified by WGS (File S2). As shown in Table 9, 45 isolates which had any drug resistance-associated mutations were susceptible by phenotypic DST. In those isolates, the following mutations were found: for INH at *ahpC*_c.-57C>T (*n* = 1); for EMB at *embB*_p.Asp328Tyr (*n* = 1), *embB*_p.Asp354Ala (*n* = 3), *embB*_p.Gln497Arg (*n* = 3), *embB*_p.Gly406Ala (*n* = 1), *embB*_p.Gly406Asp (*n* = 4), *embB*_p.Gly406Cys (*n* = 1), *embB*_p.Met306Ile (*n* = 9), *embB*_p.Met306Val (*n* = 4), and *embB*_p.Tyr319Cys (*n* = 1); for LVX at *gyrB*_p.Arg446Leu (*n* = 1), *gyrA*_p.Asp94Tyr (*n* = 1), *gyrA*_p.Asp94Gly (*n* = 1), *gyrA*_p.Ala90Val (*n* = 1), *gyrA*_p.Ala90Val + *gyrA*_p.Asp94Ala (*n* = 1), and *gyrA*_p.Ala74Ser + *gyrB*_p.Ser447Phe (*n* = 1); for MXF at *gyrA*_p.Asp94Tyr (*n* = 1), *gyrA*_p.Asp94Gly (*n* = 1), and *gyrA*_p.Ala90Val + *gyrA*_p.Asp94Ala (*n* = 1); for AMK (*n* = 1) and KAN (*n* = 1) at *rrs*_r.1401a>g; for ETH at *fabG1*_c.-8T>C (*n* = 7), *fabG1*_c.-15C>T (*n* = 3), *ethA*_c.1054_1054del (*n* = 1), *ethA*_c.341_341del (*n* = 1), *ethA*_Chromosome:g.4326004_4327421del (*n* = 1), *ethA*_p.Gln165Pro (*n* = 1), and *ethA*_p.Tyr386Cys + *fabG1*_c.-8T>C (*n* = 1); and for BDQ (*n* = 1) and CFZ (*n* = 1) at *Rv0678*_p.Arg134*.

**TABLE 8:** TB-Profiler compared with phenotypic DST in drug resistance diagnosis of MDR/RR-TB^*a*^

Drug	TB-Profiler					
	% PPV (n/N)	95% CI	% NPV (n/N)	95% CI	% consistency (n/N)	95% CI
Rifampicin	96.3 (180/187)	92.5–98.2			96.3 (180/187)	92.5–98.2
Isoniazid	95.1 (175/184)	91.0–97.4	66.7 (2/3)	20.8–93.9	94.7 (177/187)	90.4–97.1
Ethambutol	93.5 (115/123)	87.8–96.7	57.8 (37/64)	45.6–69.1	81.3 (152/187)	75.1–86.2
Levofloxacin	90.4 (75/83)	82.1–95.0	94.2 (98/104)	88.0–97.3	92.5 (173/187)	87.8–95.5
Moxifloxacin	88.6 (78/88)	80.3–93.7	97.0 (96/99)	91.5–99.0	93.0 (174/187)	88.5–95.9
Amikacin	59.1 (13/22)	38.7–76.7	99.4 (164/165)	96.6–99.9	94.7 (177/187)	90.4–97.1
Kanamycin	59.1 (13/22)	38.7–76.7	99.4 (164/165)	96.6–99.9	94.7 (177/187)	90.4–97.1
Ethionamide	51.0 (25/49)	37.5–64.4	88.4 (122/138)	82.0–92.7	78.6 (147/187)	72.2–83.9
Bedaquiline	0.0 (0/3)	0–56.2	99.5 (183/184)	97.0–99.9	97.9 (183/187)	94.6–99.2
Delamanid	0.0 (0/8)	0–32.4	100.0 (179/179)	97.9–100	95.7 (179/187)	91.8–97.8
Linezolid	0.0 (0/2)	0–65.8	100.0 (185/185)	98.0–100	98.9 (185/187)	96.2–99.7
Clofazimine	0.0 (0/1)	0–79.4	99.5 (185/186)	97.0–99.9	98.9 (185/187)	96.2–99.7

a n, number of resistant/sensitive strains predicted by TB-profiler; N, number of resistant/sensitive strains detected by phenotypic DST; PPV, positive predicted value; NPV, negative predicted value; consistency, the consistency of drug-resistant or -sensitive strains detected by TB-profiler and phenotypic DST.

**TABLE 9:** Mutations identified by TB-Profiler in phenotypically sensitive isolates

Drug	Strain	Mutation (TB-Profiler)
Isoniazid	22A188	*ahpC*_c.-57C>T
Ethambutol	22A001	*embB*_p.Gly406Asp
	22A006	*embB*_p.Gly406Asp
	22A026	*embB*_p.Met306Ile
	22A051	*embB*_p.Asp354Ala
	22A072	*embB*_p.Met306Val
	22A074	*embB*_p.Met306Val
	22A076	*embB*_p.Gly406Asp
	22A080	*embB*_p.Tyr319Cys
	22A091	*embB*_p.Met306Ile
	22A100	*embB*_p.Gln497Arg
	22A103	*embB*_p.Gln497Arg
	22A107	*embB*_p.Met306Ile
	22A109	*embB*_p.Met306Ile
	22A119	*embB*_p.Gly406Asp
	22A121	*embB*_p.Asp354Ala
	22A130	*embB*_p.Met306Ile
	22A132	*embB*_p.Asp328Tyr
	22A137	*embB*_p.Gly406Cys
	22A156	*embB*_p.Gln497Arg
	22A158	*embB*_p.Met306Val
	22A159	*embB*_p.Gly406Ala
	22A188	*embB*_p.Met306Val
	22A194	*embB*_p.Met306Ile
	22A197	*embB*_p.Met306Ile
	22A200	*embB*_p.Asp354Ala
	22A224	*embB*_p.Met306Ile
	22A227	*embB*_p.Met306Ile
Moxifloxacin	22A039	*gyrA*_p.Ala90Val, *gyrA*_p.Asp94Ala
	22A074	*gyrA*_p.Asp94Tyr
	22A145	*gyrA*_p.Asp94Gly
Levofloxacin	22A039	*gyrA*_p.Ala90Val, *gyrA*_p.Asp94Ala
	22A051	*gyrA*_p.Ala74Ser, gyrB_p.Ser447Phe
	22A064	gyrB_p.Arg446Leu
	22A074	*gyrA*_p.Asp94Tyr
	22A077	*gyrA*_p.Ala90Val
	22A145	*gyrA*_p.Asp94Gly
Kanamycin	22A081	*rrs*_r.1401a>g
Amikacin	22A081	*rrs*_r.1401a>g
Ethionamide	22A002	*fabG1*_c.-8T>C
	22A018	*ethA*_p.Tyr386Cys, *fabG1*_c.-8T>C
	22A033	*fabG1*_c.-8T>C
	22A034	*fabG1*_c.-8T>C
	22A056	*fabG1*_c.-8T>C
	22A072	*ethA*_Chromosome:g.4326004_4327421del
	22A074	*ethA*_c.1054_1054del
	22A079	*fabG1*_c.-15C>T
	22A094	*fabG1*_c.-8T>C
	22A130	*ethA*_c.341_341del
	22A140	*fabG1*_c.-8T>C
	22A145	*fabG1*_c.-15C>T
	22A153	*ethA*_p.Tyr386Cys, *fabG1*_c.-8T>C
	22A175	*ethA*_p.Gln165Pro
	22A211	*fabG1*_c.-8T>C
	22A215	*fabG1*_c.-15C>T
Bedaquiline	22A147	*Rv0678*_p.Arg134^*a*^
Clofazimine	22A147	*Rv0678*_p.Arg134^*a*^

a Stop codon.

As shown in Table 10, 56 isolates with phenotypic drug resistance had unknown drug resistance-associated mutations. Within those isolates, values were as follows: *n* = 7 for RIF, *n* = 9 for INH, *n* = 8 for EMB, *n* = 10 for MXF, *n* = 8 for LVX, *n* = 9 for AMI, *n* = 9 for KAN, *n* = 24 for ETH, *n* = 3 for BDQ, *n* = 8 for DLM, *n* = 2 for LZD, and *n* = 1 for CFZ. Next, we ran these genomes in Mykrobe to identify the relevant mutations. As illustrated in Table 10, mutations were found as follows: for INH at *katG*_p.Ser315Thr (*n* = 1); for EMB at *embB*_p.Met306Val (*n* = 2); for MXF at *gyrA*_p.Ala90Val (*n* = 1), *gyrA*_p.Asp94Ala (*n* = 1), and *gyrA*_p.Asp94Gly (*n* = 1); for KAN at *rrs*_r.1401a>g (*n* = 1), *rrs*_r.1484g>a (*n* = 1), and G in gene *eis* (*n* = 1); and for AMK at *rrs*_r.1401a>g (*n* = 2) and *rrs*_r.1484g>a (*n* = 1).

**TABLE 10:** Phenotypically resistant strains with no mutations identified by TB-Profiler

Drug	Strain	TB-Profiler	Mykrobe
Rifampicin	22A035		
	22A036		
	22A037		
	22A055		
	22A120		
	22A142		
	22A162		
Isoniazid	22A035		
	22A036		
	22A037		
	22A045		
	22A075		*katG*.p.Ser315Thr
	22A102		
	22A107		
	22A142		
	22A160		
Ethambutol	22A036		
	22A037		
	22A073		*embB*.p. Met306Val
	22A079		*embB*.p. Met306Val
	22A117		
	22A143		
	22A190		
	22A207		
Moxifloxacin	22A036		
	22A037		
	22A038		
	22A076		*gyrA*_p.Ala90Val
	22A081		*gyrA*_p.Asp94Ala
	22A103		
	22A143		
	22A170		
	22A199		*gyrA*_p.Asp94Gly
	22A214		
Kanamycin	22A037		
	22A049		
	22A082		*rrs*_r.1401a>g
	22A097		
	22A099		*rrs*_r.1484g>a
	22A121		G in gene *eis*
	22A147		
	22A148		
	22A201		
Amikacin	22A049		
	22A082		*rrs*_r.1401a>g
	22A097		
	22A099		*rrs*_r.1484g>a
	22A147		
	22A148		
	22A171		
	22A172		
	22A201		*rrs*_r.1401a>g

### Transmission networks of MDR/RR-TB.

**FIG 4:**
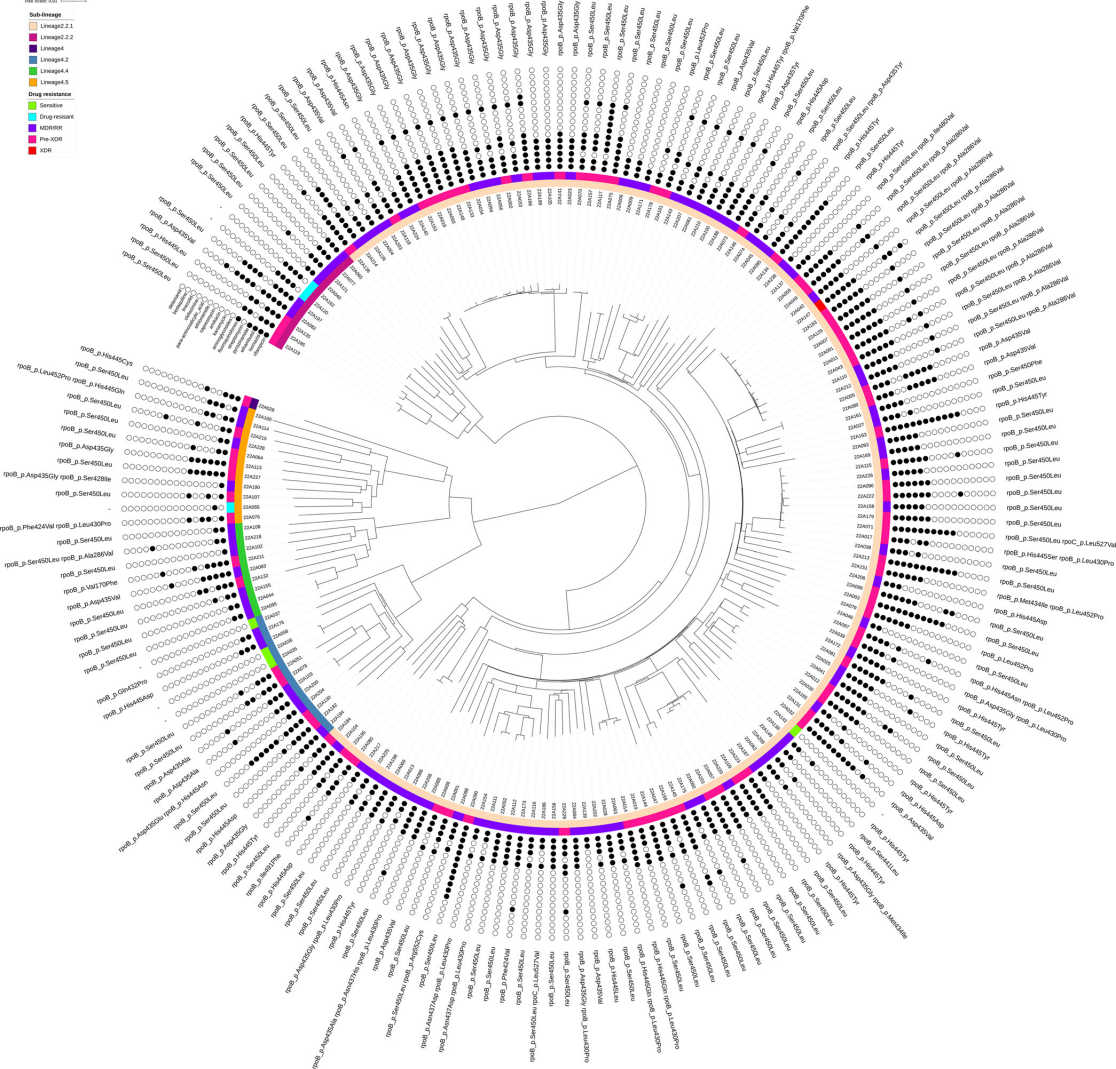
Maximum likelihood whole-genome SNP-based phylogeny of MDR/RR isolates and mutations in *rpoB* and *rpoC*. The inner ring with colors beside the nodes indicates sublineages: lineage 2.2.1 strains (bisque), lineage 2.2.2 strains (purple), lineage 4 strains (midnight blue), lineage 4.2 strains (steel blue), lineage 4.4 strains (quartz), lineage 4.5 strains (orange). The outer ring shows the presence of sensitive (lime), any drug-resistant (aquamarine), multidrug/rifampicin-resistant (MDR/RR; blue violet), pre-XDR (medium violet-red), and XDR (scarlet) genotypes among genomically clustered MDR/RR-TB strains. The black and white circles indicate the genotypic resistance to 16 antibiotics (black circle presents resistance, and white circle presents sensitivity). The *rpoB*/C mutations are annotated along the periphery of the phylogeny.

A phylogenetic tree of 187 MDR/RR-TB isolates indicating drug resistance profiles and lineages is shown in Fig. 4. A total of 152 isolates belonged to lineage 2 (East Asian), and the rest belonged to lineage 4 (Euro-American). As illustrated in Table 5, the isolates from lineage 2 were the main strains that resulted in the recent transmission of MDR/RR-TB in Chongqing.

**FIG 5:**
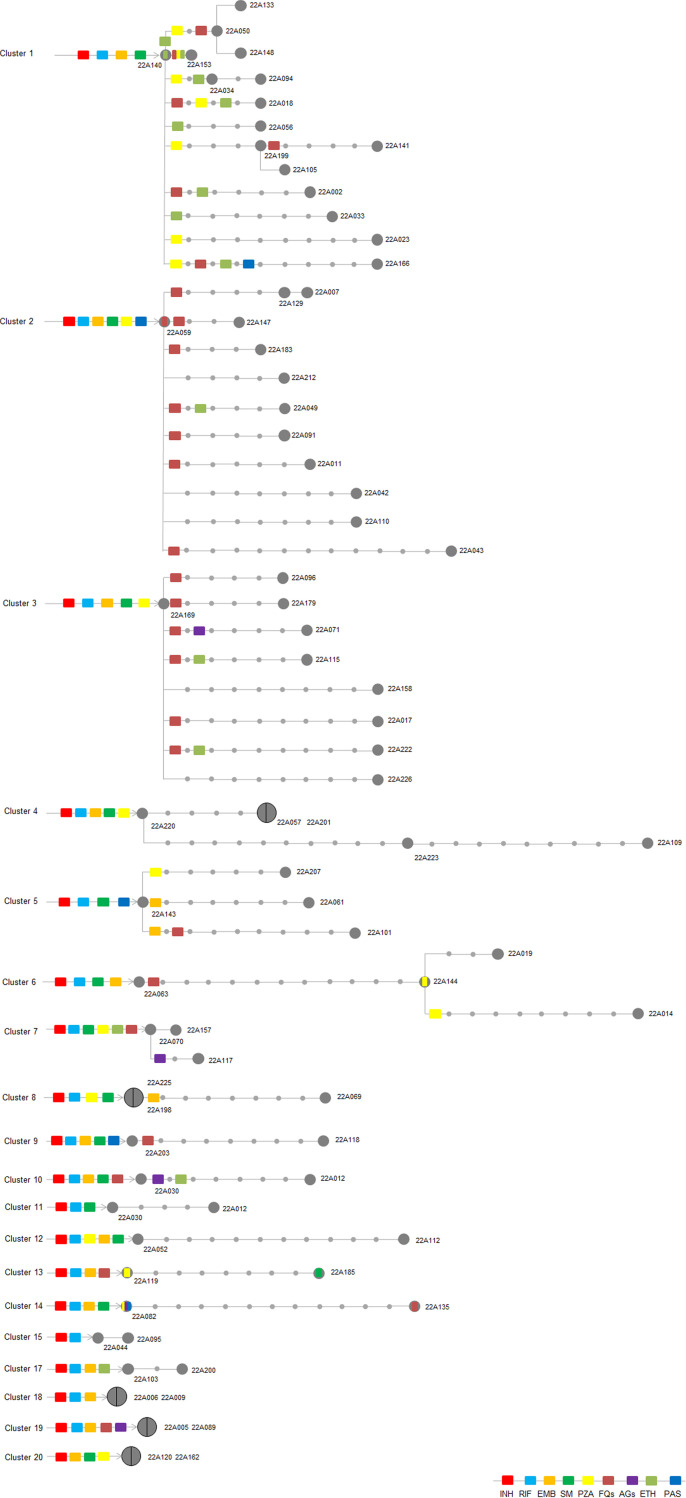
The median-joining networks for MDR/RR-TB clusters based on SNP distances and drug resistance genotypes. For each network, the arrow indicates the root and the circles represent Mycobacterium tuberculosis strains. Mycobacterium tuberculosis isolates are separated by lines, with length representing SNP distance. Isolates with identical genomes are grouped in the same circle. The colored shapes on the branches or within the circles represent resistance mutations. SM, streptomycin; PZA, pyrazinamide; FQs, fluoroquinolones; AGs, aminoglycosides; ETH, ethionamide; PAS, *para*-aminosalicylic acid.

We observed the interesting phenomenon that isolates with the same *rpoB* mutations were scattered in clusters in the phylogenetic tree (Fig. 4). To graphically certify the transmission of an MDR/RR strain, we constructed a median-joining network of each genomic cluster and mapped genotypic drug resistance onto the network (Fig. 5). The strains in 16 genomic clusters (including 2 clusters in which the strains had no *rpoB* mutations) had the same or no RIF resistance-associated *rpoB* mutations individually, except cluster 3 (one strain had *rpoB*_p.His445Ser plus *rpoB*_p.Leu430Pro, while others obtained *rpoB*_p.Ser450Leu), cluster 5 (one strain contained *rpoB*_p.Asp435Val, while others obtained *rpoB*_p.Ser450Leu), cluster 14 (the two strains had *rpoB*_p.Asp435Val and *rpoB*_p.His445Leu individually), and cluster 6 (each of 2 strains had the same mutation sites). Therefore, 76 (95.0%) of all 80 strains defined as recent transmission have the same or no mutations in the *rpoB* gene within each cluster. For INH, at least two strains shared the same mutations in individual clusters (details in File S2). These findings further proved that the resistance mutations occurred before initial transmission. Other mutations associated with drug resistance were also consistent within all strains in these 20 clusters. Fourteen clusters contained EMB resistance mutations and streptomycin (SM) resistance mutations. Taken together, the findings show that 95% (76 of 80) of genomically clustered cases of phenotypic MDR/RR-TB were from recent transmission. Fifty-five percent (44 of 80) of the MDR/RR-TB strains accumulated additional drug resistance mutations along the transmission chain, especially for FQs (63.6% [28 of 44]) (Fig. 5).

## DISCUSSION

In Chongqing, 23 of all 200 MDR/RR-TB patients died. The risk factor analysis for death showed that age was the major risk factor. Consistent with our research, MDR-TB patients aged 60 and over or with cavitary disease are more likely to have poor treatment outcomes in Zhejiang, China (23). Treatment history was the risk factor for the patients previously treated with first- or second-line drugs, and these patients were more likely to have pending outcome. Therefore, elderly patients or those previously treated with the first- or second-line drugs should have enhanced management and supervision to complete standard treatment regimens.

The control of TB transmission depends on the identification and treatment of infectious patients and their close contacts (24). In China, a setting with a high burden of TB, epidemiological data are often difficult to get. WGS can identify reasonable transmission events in patients without prior recourse to epidemiological data (5, 14, 25, 26). Among WGS analysis pipelines for detection of epidemiologically linked tuberculosis cases, the cg-MLST approach is standardized and easy to get compared to the SNP-based pipelines (27). In Chongqing, 80 (42.8%) of all 187 MDR/RR-TB patients were identified by recent transmission using a WGS-based cg-MLST approach. Patients who were infected with lineage 2 mycobacterial strains had a higher risk of recent transmission. Research regarding recent transmission of MDR strains showed that 103 (32%) of 324 MDR strains were clustered, with another 132 from treatment-naive patients; 235 (73%) patients with MDR tuberculosis probably had transmission of MDR strains in Shanghai, China (13). In our study, 21 out of all 98 genomically unique isolates were from treatment-naive patients (new cases). According to this rule, 101 (54%) patients with MDR/RR-TB probably had transmission of MDR/RR strains in Chongqing, China.

Patients with delayed diagnosis or those older than 45 years had a high risk of recent transmission of MDR-TB in Shanghai, China (13). Another study reported that the local incidence of TB in urban centers occurs via local transmission between both migrants and residents in Shanghai (28). In India, transmission of particular pre-XDR/XDR lineage 2 strains is the main driving force of the pre-XDR/XDR-TB epidemic (29). In our research, the diagnosis delay defined by the interval between the diagnosis of TB and the diagnosis of MDR/RR-TB had little effect on recent transmission of MDR/RR-TB. The local transmission between migrants and residents also had little impact on recent transmission of MDR/RR-TB. The patients with other occupations were more likely to have recent transmission in MDR/RR-TB cases. Therefore, transmission contributes the most to the MDR/RR-TB epidemic, and dominant lineage 2 strains are the main driver of the MDR/RR-TB epidemic in Chongqing, China.

In our research, TB-Profiler was used to predict the genotypic resistance (File S2). Of all 180 genotypically RIF-resistant strains, 171 (95.0%) harbored *rpoB435* (21.7% [39/180]), *rpoB445* (16.1% [29/180]), and *rpoB450* (57.2% [103/180]) mutations. The site with the most mutation was *rpoB_p.Ser450Leu*, which is consistent with other research (30). Out of the 176 genotypically INH-resistant strains, 72.7% (128/176) had *katG*, 2.3% (4/176) had *ahpC*, 2.8% (5/176) had *fabG1* (also called *inhA* promoter), 7.4% (13/176) had *ahpC* plus *katG*, and 14.8% (26/176) had *fabG1* plus *katG*. In total, 152 (86.4%) strains were observed with mutations in *katG* 315, *fabG1*_c.-15C>T, and *fabG1*_c.-8T>C. Furthermore, another study demonstrated that WGS can predict susceptibility of Mycobacterium tuberculosis to first-line drugs more accurately than phenotypic testing (15). For the new and repurposed tuberculosis drugs, no resistance mutations were observed in phenotypically BDQ-, DLM-, LZD-, and CFZ-resistant strains. The mutations associated with resistance to the new and repurposed Mycobacterium tuberculosis drugs should be updated in their resistance gene catalogue database (31–34).

The genomic clusters might represent transmission of an MDR/RR strain or initial transmission of a non-MDR/RR strain that later developed multidrug resistance (13). It is known that drug resistance of tuberculosis is mainly conferred by mutations in genes coding for drug targets or converting enzymes (35). Prior mutations may accumulate along the transmission chain, and strains may acquire new mutations that further increase drug resistance. An earlier report highlighted that both drug resistance transmission and amplification contribute to disease burden globally (36). In China, it has been proved that additional drug resistance amplified for Mycobacterium tuberculosis during the turnaround time for drug susceptibility testing (37). In Chongqing, 55% of the MDR/RR-TB strains accumulated additional drug resistance mutations along the transmission chain, especially against FQs (63.6% [28 of 44]). It is suggested that most strains developed additional drug resistance during the expansion of MDR/RR clusters, indicating that MDR/RR TB is not fully controlled. The use of a WGS-based approach for surveillance purposes might enable the public health service to take appropriate control actions in specific settings in China.

## MATERIALS AND METHODS

### Strain collection.

All suspected pulmonary tuberculosis patients are referred to local appointed hospitals, where the diagnosis is made by sputum smear and culture. For isolation from culture, each specimen was treated with 1 volume of 4% sodium hydroxide per 1 volume of sputum and then homogenized by vigorous stirring. An aliquot of 0.1 mL of the resulting specimen was inoculated into two tubes of acidified Löwenstein-Jensen (L-J) medium and incubated at 37°C. The culture was first assessed during week 1 for rapidly growing bacteria and every week for slowly growing bacteria; if no bacterial growth was observed by week 8, the result was recorded as negative. All rifampicin-resistant Mycobacterium tuberculosis isolates were previously identified using the proportion method on L-J medium containing rifampicin at a concentration of 40 μg/mL. All cases of MDR/RR-TB are reported to the Chongqing Tuberculosis Control Institute, and all culture-positive samples are delivered to the national reference laboratory for tuberculosis (NTRL) in the China CDC.

From 1 January 2018 to 31 December 2020, we enrolled 223 (24.6%) MDR/RR-TB patients from a total of 906 culture-positive TB patients in the Chongqing Tuberculosis Control Institute. The strains were thawed and subcultured on L-J medium for further analysis by combining phenotypic drug susceptibility testing (DST) and WGS at the NTRL. A total of 200 MDR/RR strains isolated from unique TB patients were used in our population-based, retrospective observational study. The strains with reculture failure and serial samples from identical patients were excluded from this study.

### Patient information collection.

Sociodemographic characteristics (gender, age, residence, and occupation) and clinical characteristics (complications and previous TB treatment history) of patients were extracted from the national drug resistance surveillance database; this information was collected and compiled by local physicians using a questionnaire filled in by patients after written informed consent was obtained at the time of patient visits.

### Strain identification.

Matrix-assisted laser desorption ionization–time of flight mass spectrometry (MADLI-TOF MS) was used to distinguish Mycobacterium tuberculosis complex (MTBC) from other mycobacteria. The detailed protocol is as follows.

Ten microliters of standard ring bacteria was harvested from L-J medium and dispersed in 1 mL of 75% alcohol (high-performance liquid chromatography [HPLC] grade), mixed well, and stored at −20°C for use. Prior to analysis, the bacterial suspension was centrifuged and the sediment was resuspended in 10 μL of acetonitrile with a small amount of zirconia/silica microbeads. Next, 10 μL of 70% formic acid was added after full-speed vortexing. The supernatant was reserved after centrifugation.

For each sample, 1 μL was deposited on a polished steel MSP 384 target plate (Bruker Daltonics, Bremen, Germany) and 1 μL of matrix solution (saturated α-cyano-4-hydroxycinnamic acid [HCCA]) was then added. The samples were air dried for 5 min before being processed in the mass spectrometer. To validate the analysis of a whole MSP 384 target, bacterial test standard (Escherichia coli DH5α protein extract) was used as a positive control and noninoculated matrix solution (HCCA) was used as a negative control. The analyses were performed using flexControl 3.0 software (Bruker Daltonics). The spectra were analyzed within an *m/z* range of 2,000 to 20,000. Four raw spectra were automatically acquired using the flexControl 3.0 software and then compared with the Bruker Daltonics database using MALDI Biotyper 3.0 software. To validate the analysis using MALDI Biotyper software, the identification of the positive control was required to be E. coli with an identification score of ≥2, and the negative control had to yield a nonidentifying score of ≤1.7.

### Risk factors of death.

In our research, all patients were classified into four groups based on the treatment outcomes when we collected the information. In the favored outcome group, the patients were cured (defined as a bacteriologically confirmed TB patient who was smear or culture negative in the last month of treatment and on at least one previous occasion during treatment) or completed treatment (defined as a bacteriologically confirmed TB patient who completed treatment without evidence of failure but with no record of a negative sputum smear or culture from the last month of treatment and at least one previous occasion during treatment); in the pending group, the patients had no treatment outcome (patients were in treatment, had an adverse reaction, refused treatment, or were lost to follow-up; the other two groups consisted of patients who died from TB and those who died from other causes. Univariate and logistic regression analyses were performed for 198 patients between each group, except for the group of death by other causes (2 patients). When presenting a risk factor analysis, we first showed the results from the univariate analysis, indicating which variables were significant (*P* < 0.1). Then, these variables were progressed to the multivariate analysis, and then we showed the results for the multivariate analysis (*P* value of <0.05 was significant).

### DST.

Drug susceptibility testing (DST) of Mycobacterium tuberculosis strains against rifampicin, isoniazid, ethambutol, kanamycin, amikacin, moxifloxacin, levofloxacin, ethionamide, bedaquiline, delamanid, linezolid, and clofazimine was performed using a UKMYC5 plate (Thermo Fisher Scientific Inc., USA), which has been reported as an alternative DST method with high accuracy and reproducibility (38). DST was conducted strictly according to the manufacturer’s instructions by trained staff at the national tuberculosis reference laboratory of China.

### Definitions.

MDR/RR-TB was defined as Mycobacterium tuberculosis resistance to at least rifampicin. MDR-TB was defined as Mycobacterium tuberculosis resistance to at least isoniazid and rifampicin. MDR-TB only was defined as an MDR-TB strain that was susceptible to both fluoroquinolones (moxifloxacin or levofloxacin) and the second-line anti-TB drugs (amikacin or kanamycin). Pre-XDR-TB was defined as MDR-TB with additional resistance to any fluoroquinolones (moxifloxacin or levofloxacin). XDR-TB was defined as MDR-TB with additional resistance to any fluoroquinolones and either BDQ or LZD.

### DNA extraction and sequencing.

We used the cetyltrimethylammonium bromide (CTAB) method to extract genomic DNA from cultures of one sputum specimen per patient. Sequencing libraries were prepared by using the Illumina Nextera kit following the manufacturer’s protocol and sequenced on the Illumina HiSeq 2500 (Illumina, San Diego, CA, USA) with 2 × 150 paired-end (PE) strategies. Coverage of 100× was expected. All whole-genome sequencing procedures were performed by Annoroad Gene Technology Company (Beijing, China). In total, we obtained WGS data for 187 strains.

### Bioinformatics analysis.

To guarantee good quality of the sequencing read, specific parameters were set and followed: 60 to 65% GC content of original data, ≤20% per-base sequence content, ≤2% overrepresented sequences, ≤10% reads containing joint sequences, ≤2% low quality reads, ≥95% of the reads mapping to reference genome, ≤20% duplicate reads, ≥95% of the reference genome being mapped by reads, ≥50× average genome sequencing depth, and ≤1% base mismatch for the reads mapping to reference genome.

We used the functionality implemented in the Ridom SeqSphere+ software (version 7.2.3) with default settings to perform cg-MLST analysis. The genome of the Mycobacterium tuberculosis strain H37Rv (GenBank accession no. NC_00962.3) served as the reference genome. Afterwards, gained genomes were compared to the seed genome to identify a list of core genome genes. Here, default settings include the removal of the shorter of two genes overlapping by more than four bases and of genes with an internal stop codon in >90% of all genomes from the scheme. cg-MLST-based neighbor joining and minimum spanning trees (MST) were calculated and drawn with SeqSphere+ software.

Lineage and sublineage calls of each isolate were made and verified using the fast-lineage-caller v1.0 (https://github.com/farhat-lab/fast-lineage-caller) (39).

A maximum likelihood SNP-based phylogeny tree was generated as we have described before (40).

Resistance genotypes were defined using the software TB-Profiler v2.8.14, available at https://github.com/jodyphelan/TBProfiler (18, 41), and Mykrobe v0.10.0, available at www.mykrobe.com (42).

### Risk factors of cluster transmission (recent transmission).

Cluster transmissions were defined as the isolates with pairwise genetic distances of fewer than 12 SNPs. Univariate and logistic regression analyses were performed for 187 patients between cluster group and noncluster group. During the presentation of a risk factor analysis, we first showed the results from the univariate analysis, indicating which variables were significant (*P* < 0.1). Then, these variables were progressed to the multivariate analysis, and then we showed the results for the multivariate analysis (*P* value of <0.05 was significant).

### Spatial-location analysis.

The residential address listed in the questionnaire of each patient was geocoded with QGIS 3.12 and Baidu Maps (Baidu, Beijing, China) to verify locations. Spatial distance indicated the distance between where two patients live on the map. Genetic distance indicated the number of different SNPs between two strains identified by the cg-MLST approach. Pearson correlation analysis between spatial distance and genetic distance (SNPs) for all 80 genomic clustered strains was performed by IBM SPSS statistics software. The greater the absolute value (0 to 1) of correlation (CRR), the stronger the correlation. Positive value is positive correlation, and negative value is negative correlation. A *P* value of <0.01 indicated significant correlation.

### Statistical analysis.

Descriptive statistics was performed for patients’ demographics and lineages, resistance categories, and clustering status of MTBC strains. Data stemming from genomic analysis of clinical isolates were analyzed statistically using IBM SPSS statistics software for Windows (version 19) and R (version 3.6.1). For univariate analysis of potential factors, we performed Fisher’s exact test. Factors of a significant result from the univariate model were included into a multivariate logistic regression analysis. Odd ratios with 95% confidence interval (CI) were estimated, and variables with *P* values of less than 0.05 were taken as significant predictors.

### Ethics statement.

The institutional review boards of the China CDC approved the study. All patients provided written informed consent.

### Data availability.

The accession numbers of all sequenced genomes from this study are shown in File S4.
