# Supplementary material for: Transmission and Drug Resistance Genotype of Multidrug-Resistant or Rifampicin-Resistant Mycobacterium tuberculosis in Chongqing, China
Source: Microbiol Spectr. 2022 Oct 10;10(5):e02405-21. doi: 10.1128/spectrum.02405-21 (PMC9604020; doi:10.1128/spectrum.02405-21)
Supplement: Supplemental file 5 — Download spectrum.02405-21-s0005.pdf, PDF file, 3.7 MB [file spectrum.02405-21-s0005.pdf]

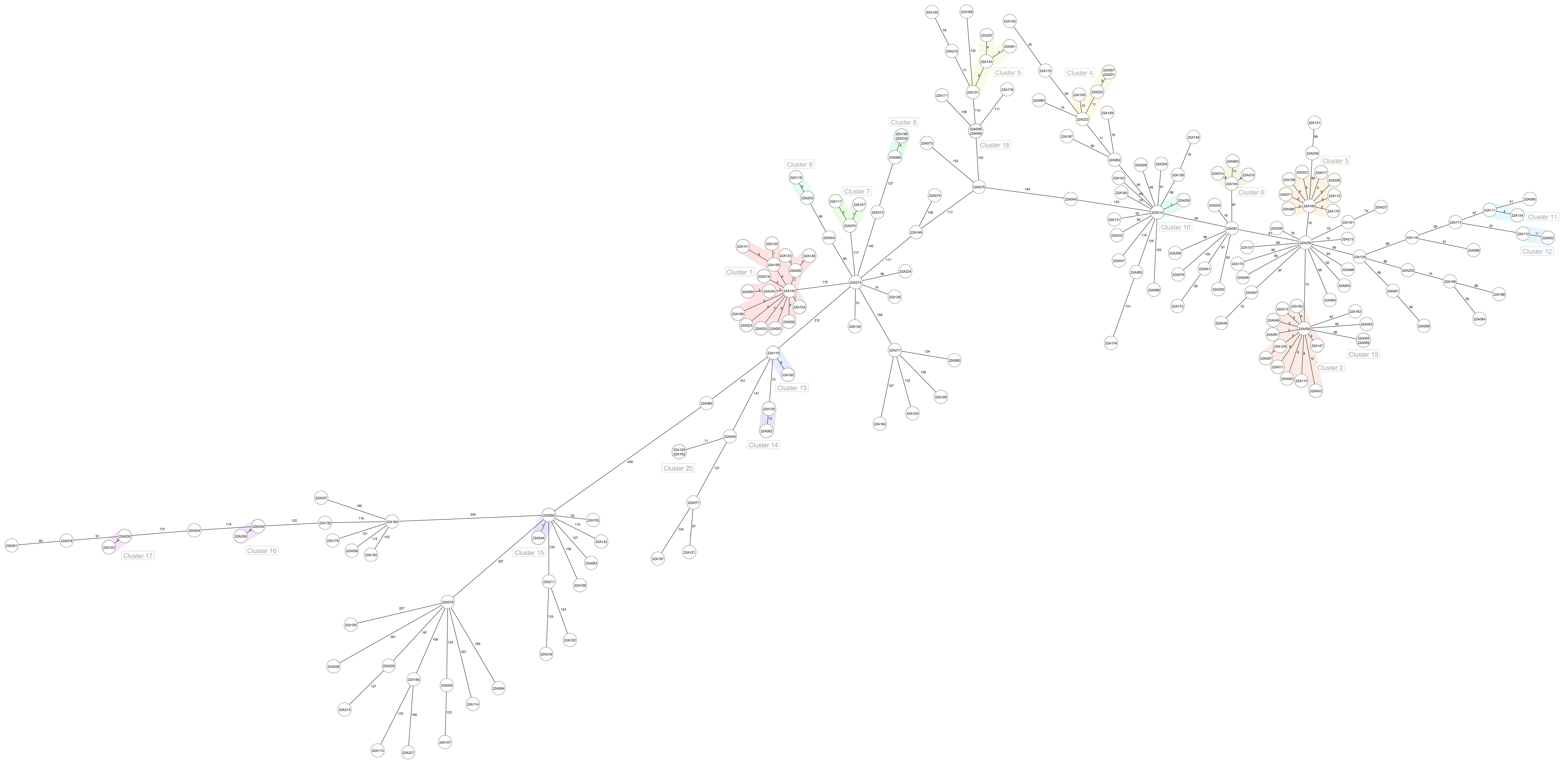
Cluster 1
Cluster 2
Cluster 3
Cluster 4
Cluster 5
Cluster 6
Cluster 7
Cluster 8
Cluster 9
Cluster 10
Cluster 11
Cluster 12
Cluster 13
Cluster 14
Cluster 15
Cluster 16
Cluster 17
Cluster 18
Cluster 19
Cluster 20

Supplemental figure legends:

Figure S1: Minimum distance spanning tree (MST) of MDR/RR strains based on cg-MLST typing. Cluster strains that differed by 12 or fewer allele thresholds showed in different colors.
